# Dummy-run for standardizing plan quality of intensity-modulated radiotherapy for postoperative uterine cervical cancer: Japan Clinical Oncology Group study (JCOG1402)

**DOI:** 10.1186/s13014-019-1340-y

**Published:** 2019-07-29

**Authors:** Hiroyuki Okamoto, Naoya Murakami, Fumiaki Isohashi, Takahiro Kasamatsu, Yoko Hasumi, Kotaro Iijima, Shie Nishioka, Satoshi Nakamura, Mitsuhiro Nakamura, Teiji Nishio, Hiroshi Igaki, Yuko Nakayama, Jun Itami, Satoshi Ishikura, Yasumasa Nishimura, Takafumi Toita

**Affiliations:** 1 Department of Medical Physics, National Cancer Center Hospital, 5-1-1 Tsukiji, Chuo-ku, Tokyo, 104-0045 Japan; 2 Department of Radiation Oncology, National Cancer Center Hospital, Tokyo, 104-0045 Japan; 3 Department of Radiation Oncology, Graduate School of Medicine, Osaka University, Osaka, 565-0871 Japan; 4 Department of Obstetrics and Gynecology, Tokyo Metropolitan Bokutoh Hospital, Tokyo, 130-8575 Japan; 5 Department of Obstetrics and Gynaecology, Mitsui Memorial Hospital, Tokyo, 101-8643 Japan; 6 Department of Information Technology and Medical Engineering, Human Health Science, Graduate School of Medicine, Kyoto University, Kyoto, 606-8507 Japan; 7 Department of Medical Physics, Graduate School of Medicine, Tokyo Women’s Medical University, Tokyo, 162-8666 Japan; 8 Department of Radiology, Graduate School of Medical Sciences, Nagoya City University, 1 Kawasumi, Mizuho-cho, Mizuho-ku, Nagoya, Aichi 467-8601 Japan; 9 Department of Radiation Oncology, Kindai University Faculty of Medicine, 377-2 Ohno-Higashi, Osaka-Sayama, Osaka, 589-8511 Japan; 10 Radiation Therapy Center, Okinawa Chubu Hospital, Okinawa, 904-2293 Japan

**Keywords:** Cervical cancer, IMRT, VMAT, Dummy-run

## Abstract

**Background:**

The purpose of this study was to assess compliance with treatment planning in a dummy-run for a multicenter clinical trial involving patients with high-risk postoperative uterine cervical cancer using intensity-modulated radiation therapy (IMRT) (JCOG1402 trial).

**Methods:**

For the dummy-run, we prepared a computed tomography dataset comprising two anonymized cases of post-hysterectomy cervical cancer. These were sent to the 47 participating institutions to assess institutional plan quality such as delineations and dose distributions.

**Results:**

Central review showed 3 and 4 deviations per treatment plan on average. The deviations related to the nodal and vaginal cuff clinical target volume (CTV) delineation, which accounted for approximately 50% of the total deviations. The CTV vaginal cuff showed considerable differences in delineation compared with the nodal CTV. For the Dice similarity coefficient, case 1 showed a mean ± 1σ of 0.81 ± 0.03 and 0.60 ± 0.09 for the nodal and the CTV vaginal cuff, respectively, while these were 0.81 ± 0.04 and 0.54 ± 0.14, respectively, for case two. Of the 47 institutions, 10 were required to resubmit their treatment plan because the delineations, planning target volume margin, and required dose distributions were not in accordance with the JCOG1402 protocol.

**Conclusions:**

The dummy-run test in postoperative uterine cervical cancer demonstrated substantial deviations in the delineations, particularly for the CTV vaginal cuff. The analysis data could provide helpful information on delineation and planning, allowing standardization of IMRT planning for postoperative uterine cervical cancer.

**Trial registration:**

Japanese Clinical Trial Registry #: UMIN000027017 at https://upload.umin.ac.jp/cgi-open-bin/ctr/ctr_view.cgi?recptno=R000030672;language=J

**Electronic supplementary material:**

The online version of this article (10.1186/s13014-019-1340-y) contains supplementary material, which is available to authorized users.

## Background

The use of intensity-modulated radiation therapy (IMRT) for post-operative patients with uterine cervical cancer allows for a reduction in unwanted doses to healthy organs [1–4] and radiation-related complications [5, 6], compared with three-dimensional conformal radiotherapy (3DCRT). For instance, some studies reported lower doses to the bladder, rectum, and bowel with IMRT than with 3DCRT [1, 2]. Roeske et al. reported that the bowel dose in IMRT could be reduced by approximately 50% compared with that in 3DCRT [1]. The NRG Oncology/RTOG 1203 trial aimed to assess acute toxicity and quality of life during treatments with 3DCRT and IMRT for patients with cervical and endometrial cancer. In 2018, it was reported that IMRT has significantly less gastrointestinal (GI) and urinary toxicity than 3DCRT [7].

In 2017, the Japan Clinical Oncology Group (JCOG) started a multicenter clinical trial for high-risk postoperative patients with uterine cervical cancer using IMRT, called JCOG1402 [8, 9]. The primary endpoint is to confirm the non-inferiority of concurrent chemoradiotherapy using IMRT compared with the historical control data of 3DCRT-CCRT [10] in terms of 3-year relapse-free survival (RFS). The secondary endpoints are overall survival, local RFS, proportion of ≥ grade 3 late lower GI toxicity, proportion of limbs with edema (lower), adverse events, and serious adverse events.

According to the JCOG1402 protocol [9], participating institutions shall meet the following requirements for credentials before enrolling the patients: (i) the institution has performed whole-pelvis IMRT for more than five patients in clinical practice; (ii) institutional treatment accuracy shall be confirmed to be within a 3% dose difference and positional differences of 3 mm for the radiation field, which is independently assessed by the Medical Physics Working Group (MPWG) of the Radiation Therapy Study Group (RTSG) in the JCOG [11, 12]; and (iii) dummy-run: the institution shall submit two cases of treatment planning in postoperative uterine cervical cancer. These treatment plans are assessed by central review to ensure they are appropriate for IMRT treatment planning.

Our purpose is to assess the compliance with treatment planning in a dummy-run for JCOG1402, and the dummy-run in this study will provide helpful information on delineation and planning for the participating institutions in order to standardize IMRT planning for postoperative uterine cervical cancer.

## Methods

### Protocol design

A working group (WG) on postoperative IMRT for uterine cervical cancer was formulated in the RTSG of the JCOG in April 2013. The WG collected information on the clinical concerns about treatment protocols, delineation, immobilization, optimization techniques, prescribed dose, required dose distribution, patient setup, and bladder volume management, and held extensive discussions about establishing an IMRT protocol for a prospective clinical trial JCOG1402 [13]. The protocol was described as follows. The clinical target volumes (CTV) and organ at risks (OARs) in the JCOG1402 protocol were to be delineated according to the CTV contouring guidelines [14, 15], and RTOG guidelines of the OARs [16]. The 1402 protocol contains the pictorial atlas for the CTVs as well as the OARs. The CTV includes CTV vaginal cuff and paracolpium and subclinical lymph node CTV. The bowel bag, an imaginary structure that resembles the peritoneal cavity and was used to surrogate bowel dose, was delineated instead of the actual bowel loop because assessing the dose to the bowel loop itself is challenging owing to internal organ movement during treatment fractions. The vagina cuff planning target volume (PTV) margin will be adequate to cover the geometrical uncertainty due to large inter-fractional movements. According to our previous report [17], a geometrical margin was proposed to define the vaginal cuff PTV; 0.5, 1.0, and 1.5 cm in the right–left (RL), superior–inferior (SI), and anterior–posterior (AP) direction, respectively, based on full bladder computed tomography (CT) images. Management of the daily bladder volume is essential to minimize inter-fractional variations in bladder volumes or vaginal cuff movements. In addition, a full bladder preparation pushes the small bowel away from the treated region within the pelvis, which results in reduction of the bowel dose. A dose covering 50% of the PTV of 50.4 Gy in 28 fractions was applied to both the nodal and the vaginal cuff PTV. The JCOG1402 dose and dose-volume criteria were derived with consideration of variations of dosimetric parameters in IMRT from the five representative domestic institutions and expert opinions (Table 1).

**Table 1:** Dose and dose-volume criteria for the target volumes and organs at risk. 100% indicates the prescribed dose of 50.4 Gy

Structure	Structure name		Per protocol	Acceptable variation
Body		Global D_max_	< 115%	<120%
PTV	PTV	D_50%_	=100%	–
		D_98%_	>90%	>85%
		D_95%_	>95%	>90%
		D_2%_	<110%	<115%
Overlap between PTV and bowel bag	OL_PTV_Bowel	D_max_	<105%	<110%
Rectum	Rectum	V_40 Gy_	<85%	<95%
		V_50 Gy_	<40%	<60%
		D_max_	<110%	<120%
Bladder	Bladder	V_45 Gy_	<50%	<70%
		D_max_	<110%	<120%
Bowel bag	Bowel bag	V_40 Gy_	<40%	<50%
Pelvic bones	Pelvic bones	V_10 Gy_	<85%	<95%
		V_40 Gy_	<30%	<50%
Femoral joint	Femoral joint	V_30 Gy_	<40%	<60%

Abbreviations: *D*_*max*_ the maximum dose, *D*_*x%*_ dose covering x% of the volume of the organ, *V*_*y Gy*_ volume receiving y Gy

**Fig. 1:**
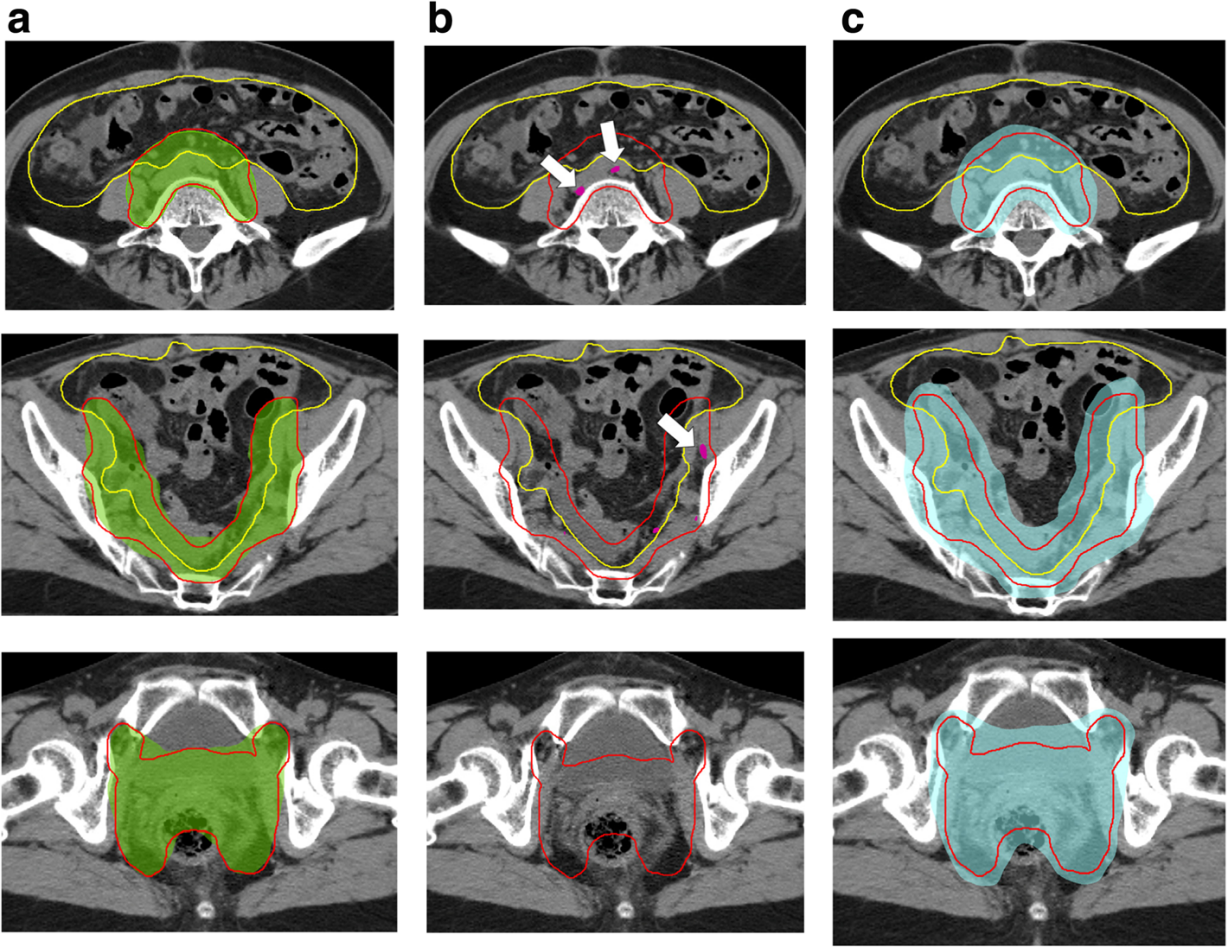
JCOG1402 protocol guidelines for ideal dose distributions at a dose level of (**a**) 95% (green), (**b**) 105% (pink), and (**c**) 40 Gy (cyan) with the vaginal cuff and nodal PTV (red)

As shown Fig. 1, the WG also discussed the ideal dose distribution for the three dose levels: (a) 95%, (b) 105%, and (c) 40 Gy. The 95% dose level should cover the whole PTV, and a cold spot less than 95% should not develop inside the primary lesion, i.e., the vaginal cuff, to prevent vaginal recurrence because 3DCRT achieves an excellent uniform dose inside the target volume. Regarding toxicity, 105% of the prescribed dose inside the bowel, including the overlap region of the PTV and the bowel bag, should be avoided. Additionally, the bowel and bladder should be spared to form a horseshoe shape at a dose level of 40 Gy. Information such as the required dose distributions, was provided to the participating institutions for dummy-run tests. The patient setup technique was based on bony structures, and cone-beam CT scans were performed to assess the inter-fractional displacements in the bladder, rectum, and bowel.

The clinical trial JCOG1402 was approved by the Institutional Ethical Review Board of the National Cancer Center Hospital (Approval number: 2015–359) and was performed in accordance with the ethical standards stipulated in the 1964 Declaration of Helsinki and its later amendments.

### Dummy-run

We prepared two anonymized typical cases from the post-hysterectomy cervical cancer CT dataset from the 1st author’s institution. Both cases had vaginal markers for the definition of the CTV vaginal cuff, and case 2 had a moderate amount of ascites. The anonymized CT dataset was then sent to the 47 institutions, and the delineation and IMRT planning were conducted according to the JCOG1402 protocol. The 47 institutions conducted IMRT plans (delineations and dose calculations) for two cases while referring to the JCOG 1402 protocol including the pictorial atlas and the dose constraints. Between November 2016 and June 2018, dummy-run meetings were held eight times, 94 treatment plans including the two cases sent to the 47 institutions were assessed via a central review by the JCOG1402WG. Regarding assessment of inter-observer variation for delineations of the CTV, MIM maestro (MIM Software Inc., OH, USA) was used to calculate the Dice similarity coefficient (DSC) [18] and the Hausdorff distance (HD) [19].

The assessed categories in the central review are summarized in the Additional file 2: Table S1. These are categorized as follows: (1) Delineation: (a) the nodal and (b) CTV vaginal cuff (c) CTV–PTV margin, and (d–f) rectum, bowel bag, and pelvic bones; (g) dose distribution; and (h) dose and dose-volume criteria for each structure. The definition of major deviations in this dummy-run was determined to be deviations from delineation of the nodal and CTV vaginal cuff and/or the ideal dose distribution. If major deviations are observed, the institutions will be required to revise and improve the treatment plans.

## Results

### Dummy-run

**Fig. 2:**
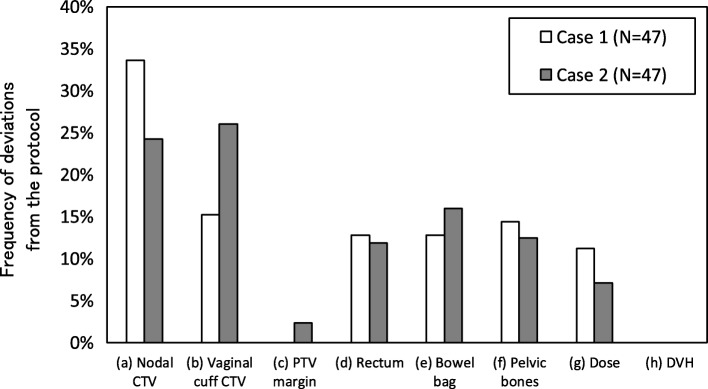
Categories of the obtained deviations from the protocol for 94 treatment plans including two cases from the 47 institutions

Figure 2 shows the numbers of deviation as a function of the categories. The total number of observed deviations for case 1 and 2 was 125 and 169, respectively. On average, 3 and 4 deviations per treatment plan were determined. The deviations related to the CTV delineation accounted for approximately 50% of the total deviations. Table 2 shows details of the total deviations shown in Fig. 2. For nodal CTV, significant deviation in the caudal direction was observed in two cases. In the conventional 3DCRT planning, field edges are created with multi-leaf collimators based on the bony structure. Delineation of the obturator lymph node area near the superior part of the obturator foramen may not be familiar to physicians in some institutions. For CTV vaginal cuff, frequent deviation was observed in the anterior, posterior, cranial, and lateral borders, while less deviation was noticed in the caudal direction. The most frequent deviation was inadequate anterior margins of the CTV vaginal cuff. In such cases, the anterior margin should have been at the posterior border of the bladder or retropubic pad of fat. In some cases, the posterior border of the CTV vaginal cuff seemed inappropriate, and it should have been the anterior border of the mesorectal fascia or anterior wall of the rectum. Some institutions did not measure distance from the vaginal marker/gauze, as noted in the Additional file 2: Table S1. An unnecessarily large caudal margin with a maximum difference of 2.4 cm was observed for one institution.

**Table 2:** Gathering major pitfalls of two cases among 94 treatment plans from the 47 institutions through the dummy-run test

Item	No. of deviations	Contents (*N* = 94)
Nodal CTV		
27		Caudal margin of the obturator lymph node area is the superior part of the obturator foramen.
14		Caudal margin of the presacral lymph node area is the lower level of S2 or cranial section of the piriform muscle.
13		Adipose connective tissue between lateral surface of vertebral body and psoas muscle is included in the CTV.
13		Caudal margin of the external iliac lymph node area is the superior aspect of the femoral head.
5		Cranial margin of the common iliac lymph node area is aortic bifurcation.
2		Bone or muscle is excluded from the CTV.
9		Others
CTV vaginal cuff		
19		Anterior margin of the CTV vaginal cuff is the posterior border of the bladder or retropubic pad of fat.
16		Posterior margin of the CTV vaginal cuff is the anterior part of the mesorectal fascia or anterior wall of the rectum.
13		Cranial margin of the CTV vaginal cuff is 1–1.5 cm cranial from the most cranial vaginal marker/gauze.
11		Lateral margin of the CTV vaginal cuff is the medial edge of the internal obturator, piriformis, coccygeus, iliococcygeus, or puborectalis muscle; the ishiorectal fossa should be excluded from the CTV vaginal cuff.
4		Caudal margin of the CTV vaginal cuff is 3–4 cm caudal from the most cranial vaginal marker/gauze or at lowest level of the obturator foramen, whichever is lower.
Bowel bag		
9		Cranial margin in the protocol was not adhered to, leading to inaccuracy of dosimetric evaluation.
7		A part of the rectum was included.
7		Caudal margin was inadequate, and it should be the lowest part of the bowel.
3		Bladder should be excluded from the bowel
17		Others
f) Pelvic bone		
13		Delineation region in the protocol was not adhered to, leading to inaccuracy of dosimetric evaluation.
10		Bone marrow and intervertebral disc were not partially included, leading to inaccuracy of dosimetric evaluation for hematotoxicity.
16		Others
g) Dose distribution		
10		Insufficient 95% dose coverage.
6		Insufficient 105% dose coverage.
10		Insufficient dose sparing of 40 Gy.

**Fig. 3:**
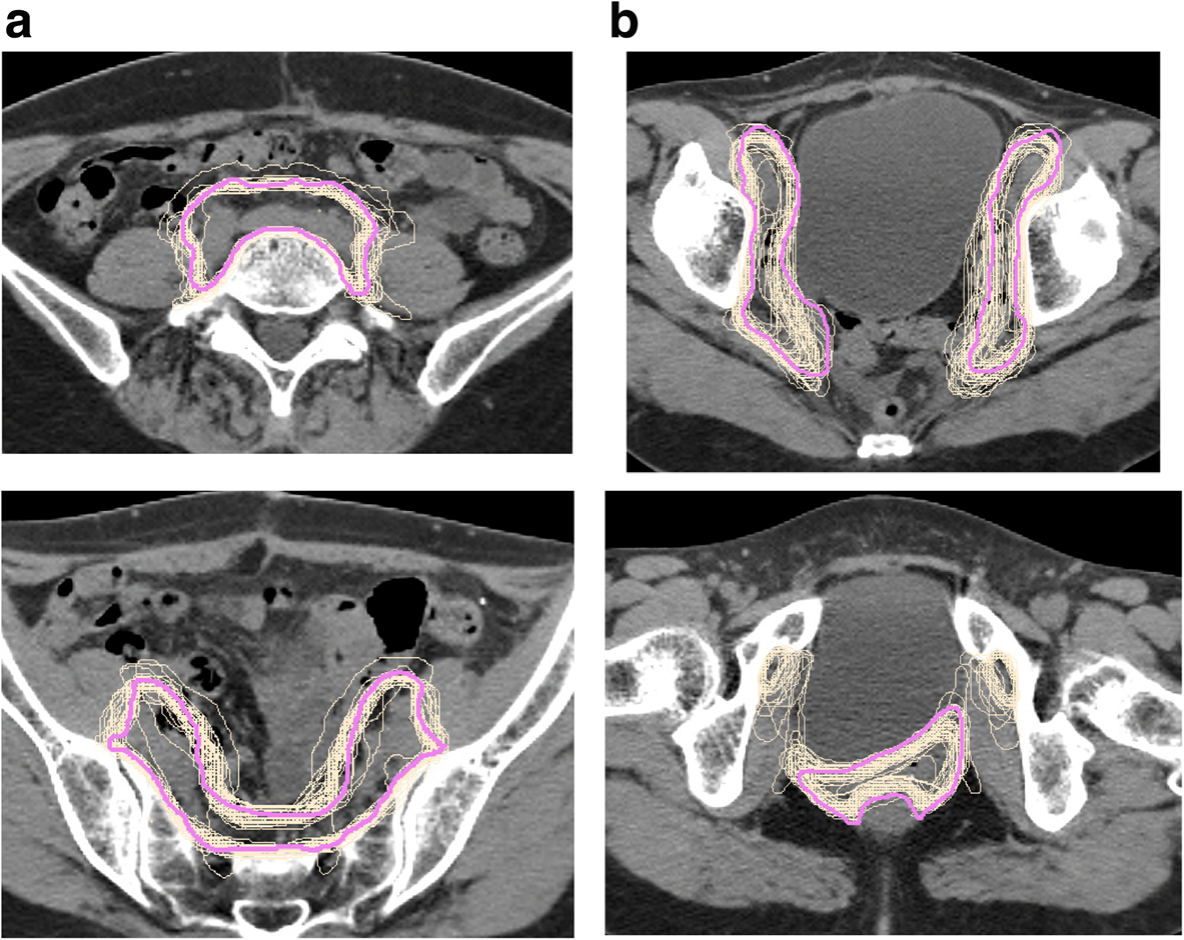
CTVs for (**a**) nodal and (**b**) vagina cuff from the 47 institutions in case 1. The reference delineation is shown in pink

**Fig. 4:**
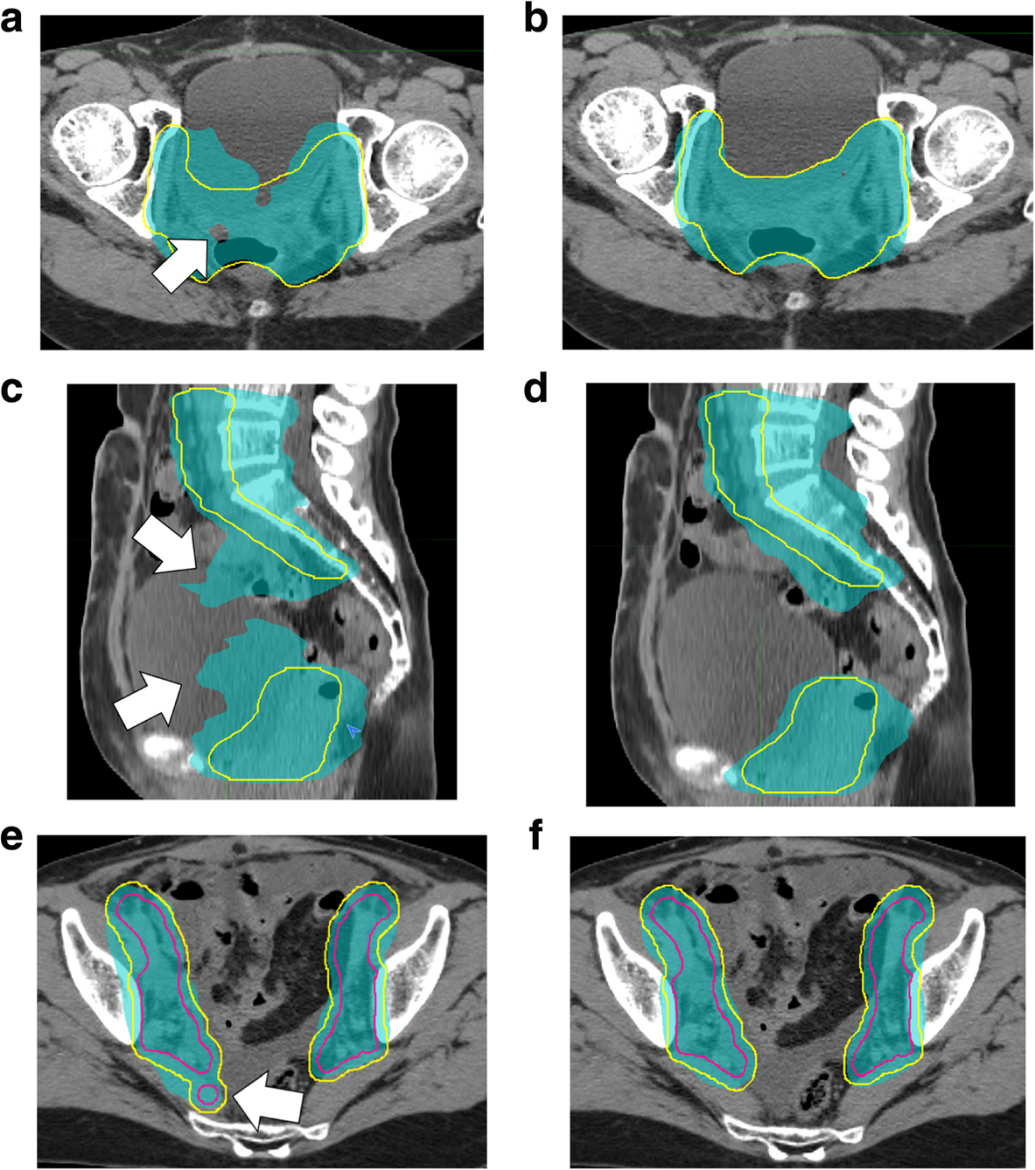
Revised treatment plan (**b**, **d**, **f**) for the (**a**) emergence of a cold spot (≤95% dose). (**b**) broadening of 40 Gy in the bowel and the bladder region, and (**e**) wrong PTV due to the presence of tiny structures resulting from an operational mistake in delineations with a color wash at a 95% dose level, PTV(yellow), and CTV (pink, only Fig. 4e and f)

Figure 3 shows the 47 CTVs in case 1, with the reference delineations (pink). For case 1, the mean ± 1σ of the DSC for the nodal and CTV vaginal cuff was 0.81 ± 0.03 (range: 0.68–0.86) and 0.60 ± 0.09 (range: 0.42–0.79), respectively. The HD with a unit of mm for the nodal and CTV vaginal cuff was 18.6 ± 6.3 (range: 10.9–32.6) and 24.7 ± 6.0 (range: 10.6–37.9), respectively. For case 2, the mean ± 1σ for the DSC was 0.81 ± 0.04 (range: 0.71–0.87) and 0.54 ± 0.14 (range: 0.23–0.78), respectively. The HD for the nodal and CTV vaginal cuff was 21.6 ± 10.4 (range: 12.3–83.4) and 32.4 ± 13.5 (range: 14.5–56.0), respectively. All treatment plans were finally accepted, with resubmissions required for 10 of the 47 institutions, although 11 major deviations were observed. Notably, seven cases were found to have a major deviation of delineation, particularly for the CTV vaginal cuff, or the submitted dose distributions did not meet the protocol requirements. For instance, a cold spot emerged inside the primary lesion (Fig. 4a). In the revised treatment plan (Fig. 4b), adequate dose coverage to the PTV was achieved. A broadening of 40 Gy in the bowel and the bladder region was also noted (Fig. 4c). In the revised treatment plan (Fig. 4d), these organs were spared from the 40 Gy dose. In another two cases, the PTV margin did not comply with the protocol. The two remaining cases had an incorrect PTV due to the presence of tiny structures in the nodal CTV (Fig. 4e). This might have been caused by an operational mistake in delineations, which results in an unnecessary treated region, as indicated by the white arrow in the figure. The color wash was displayed with the 95% dose level. In the revised plan (Fig. 4f), the PTV and dose distributions were corrected by erasing the tiny structure in the CTV. Additional file 1: Figure S1 shows the boxplots for the dose-volume histogram (DVH) results for 94 treatment plans in each case with the dose and dose-volume criteria (triangle marker). As shown in the figure, the D_98%_ and D_95%_ of the PTV was greater than the required criteria “per protocol,” and the dose coverage of PTV in IMRT could be achieved. Additionally, almost all of the institutions succeed in preventing emergence of high doses to the bowel, including the overlap region of the PTV. This was because the upper quartile of the maximum dose in this region was lower than the global maximum dose. Additionally, it was observed that the lower quartile in almost all of the critical organs could be lower than “per protocol”.

## Discussion

The dummy-run in clinical trials is essential to ensure the quality of the study and to provide opportunities for early understanding of the protocol concept for participating institutions. Continuing education is important, and a dummy-run can help determine misinterpretations of the protocol before patient enrollment. Dummy-run studies have been reported from the Radiation Therapy Oncology Group (RTOG) and the European Organisation for Research and Treatment of Cancer (EORTC) [20–25]. For instance, variations in the target volume and OAR definitions in head and neck cancer, lung, and prostate cancer were observed [20–23]. Generally, they concluded that a strict quality assurance procedure should be followed for radiotherapy. Meanwhile, only performing a dummy-run is inadequate for quality assurance (QA), although this is a limitation of this study. It is emphasized that individual case reviews are also encouraged to observe protocol variations [24, 25].

Regarding a dummy-run using the common CT dataset, the inter-observer error for delineations can be quantitatively evaluated. Eminowicz et al. demonstrated wide inter-observer variations in delineation of the CTV for definitive radiotherapy in cervical cancer [26] and its dosimetric impact [27]. In this study, the DSC and HD were used to quantitatively evaluate deviations of delineations from the reference. These metrics have been widely used as a standard tool. The DSC can be derived from the relative overlap area, while the HD can be calculated to quantitatively evaluate maximum gross deviations in surface dimensions. These metrics in this study showed that the DSC in the CTV vaginal cuff was lower than that in the nodal CTV for both cases. Additionally, the DSC for case 2 was lower than that for case 1. Similarly, the HD in the CTV vaginal cuff was greater than the nodal CTV. There are several possible reasons for the poorer agreement in the CTV vaginal cuff than in the nodal CTV. Some institutions did not notice small metallic markers inserted in the vaginal vault, and case 2 had moderate amount of ascites that made it difficult to define the CTV vaginal cuff. Meanwhile, because the nodal CTV is generally determined by the vascular structures, muscle, and bone, it is easier to delineate the nodal CTV than the CTV vaginal cuff.

In our previous study [17], the CTV vaginal cuff showed greater inter-fractional variations in the SI and AP direction than RL. For instance, the 95th percentile of uncertainty of vaginal clips in RL, SI, and AP was 0.3, 0.7, and 1.2 cm, respectively. In this, study, it was observed that the vaginal cuff with a high risk of local recurrence had large uncertainties in delineations. Therefore, not only an adequate PTV-CTV margin, but also accurate delineations in the vaginal cuff is indispensable to prevent local recurrences in IMRT. The dummy-run test can effectively achieve the goal.

The JCOG1402 dose and dose-volume criteria were appropriate to achieve clinically acceptable treatment plans. These criteria could be established from the collected data using the DVH from the five representative institutions. In the process of developing the criteria, the previously published compliance data of the RTOG 0418 [28] were taken into account. i.e., the criteria in rectum and bladder were relaxed in the JCOG1402. Additionally, these criteria were modified by the clinical perspectives as follows: the secondary endpoint of JCOG1402 is to show the reduction of GI toxicity compared with 3DCRT historical control data. Therefore, a high dose in the bowel should be avoided, e.g., the maximum dose in the whole bowel bag, including the overlap of the PTV, should be less than 110% of the prescribed dose (Table 1). Moreover, a median dose is applied to the prescribed dose in the PTV. Therefore, dose coverage to the PTV should be carefully applied. In particular, the cold spot within the CTV vaginal cuff should be avoided because the lesion is considered as having a high risk of recurrence. A cold spot is possibly caused by strict dose constraints in the critical structures in optimization. Thus, not only constraint of D_98%_ and D_95%_ in the PTV, but also the ideal dose distributions (Fig. 1a) are provided in this protocol to retain a dose coverage equivalent to that in 3DCRT. A dose level of 40 Gy must be spared in the bowel region (Fig. 1c). Isohashi et al. reported that V_15–45 Gy_ in the small bowel loops had high accuracy to predict GI complications, and multivariate analysis indicates V_40 Gy_ in the small bowel loops as an independent predictor of chronic GI complications [5]. By providing not only the dose and dose-volume criteria, but also the ideal dose distribution for the 95, 105%, and 40 Gy dose level to participating institutions, we could effectively standardize the treatment quality in the JCOG1402 protocol.

The dummy-run demonstrate trends of deviation from the protocol, and also provided information which could lead to standardizing plan quality in the JCOG 1402. Such helpful information has been fed back to participating institutions through specific face-to-face meetings which were held eight times between November 2016 and June 2018. In addition, the QA criteria established from the dummy-run process are used in the individual case reviews for patients entered in the JCOG1402.

## Conclusions

The dummy-run test in postoperative uterine cervical cancer demonstrated substantial deviations in the delineations, particularly for the CTV vaginal cuff. The analysis data could provide helpful information on delineation and planning, allowing standardization of IMRT planning for postoperative uterine cervical cancer.

## Additional files

Additional file 1: **Figure S1**. DVH criteria and the box-plots with a bar of range for 94 treatment plans in two cases: Case 1: (a) and (b), Case 2: (c) and (d). 100% of dose and volume means the prescribed dose of 50.4 Gy and a whole volume of a structure, respectively (DOCX 46 kb)

Additional file 2: **Table S2.** Assessed categories in the central review. (DOCX 16 kb)

## Data Availability

Not applicable.

## Abbreviations

3DCRT: Three-dimensional conformal radiotherapy
CCRT: Concurrent chemoradiotherapy
CT: Computed tomography
CTV: Clinical target volume
D_max_: Maximum dose
D_x%_: Dose covering x% of the volume of the organ
EORTC: European Organisation for Research and Treatment of Cancer
GI: Gastrointestinal
IMRT: Intensity-modulated radiation therapy
JCOG: Japan Clinical Oncology Group
MPWG: Medical Physics Working Group
OAR: Organ at risks
PTV: Planning target volume
QA: Quality assurance
RTOG: Radiation Therapy Oncology Group
RTSG: Radiation Therapy Study Group
V_y Gy_: Volume receiving y Gy
WG: Working group

## References

[1] Roeske JC, Lujan A, Rotmensch J (2000). Intensity-modulated whole pelvic radiation therapy in patients with gynecologic malignancies. Int J Radiat Oncol Biol Phys.

[2] Ahamad A, D'Souza W, Salehpour M (2005). Intensity-modulated radiation therapy after hysterectomy: comparison with conventional treatment and sensitivity of the normal-tissue-sparing effect to margin size. Int J Radiat Oncol Biol Phys.

[3] Mell LK, Tiryaki H, Ahn KH (2008). Dosimetric comparison of bone marrow-sparing intensity-modulated radiotherapy versus conventional techniques for treatment of cervical cancer. Int J Radiat Oncol Biol Phys.

[4] Murakami N, Okamoto H, Kasamatsu T (2014). A dosimetric analysis of intensity-modulated radiation therapy with bone marrow sparing for cervical cancer. Anticancer Res.

[5] Isohashi F, Yoshioka Y, Mabuchi S (2013). Dose-volume histogram predictors of chronic gastrointestinal complications after radical hysterectomy and postoperative concurrent nedaplatin-based chemoradiation therapy for early-stage cervical cancer. Int J Radiat Oncol Biol Phys.

[6] Chopra S, Dora T, Chinnachamy AN (2014). Predictors of grade 3 or higher late bowel toxicity in patients undergoing pelvic radiation for cervical cancer: results from a prospective study. Int J Radiat Oncol Biol Phys.

[7] Klopp AH, Yeung AR, Deshmukh S. Patient-reported toxicity during pelvic intensity-modulated radiation therapy: NRG oncology–RTOG 1203. J Clin Oncol. 2018. 10.1200/JCO.2017.77.4273 PMC6097832 29989857

[8] Murakami N, Isohashi F, Hasumi Y, et al. Single-arm confirmatory trial of postoperative concurrent chemoradiotherapy using intensity modulated radiation therapy for patients with high-risk uterine cervical cancer:Japan clinical oncology group study (JCOG1402). Jpn J Clin Oncol, accepted. 2019. 10.1093/jjco/hyz098 31613355

[9] The Japan Clinical Oncology Group (JCOG) 1402. https://upload.umin.ac.jp/cgi-open-bin/ctr/ctr_view.cgi?recptno=R000030672;language=J [accessed 27 June 2019].

[10] Isohashi F, Takano T, Onuki M (2019). A multi-institutional observational study on the effects of three-dimensional radiotherapy and weekly 40-mg/m2 cisplatin on postoperative uterine cervical cancer patients with high-risk prognostic factors. Int J Clin Oncol.

[11] Nakamura M, Minemura T, Ishikura S (2016). An on-site audit system for dosimetry credentialing of intensity-modulated radiotherapy in Japanese clinical oncology group (JCOG) clinical trials. Phys Med.

[12] Okamoto H, Minemura T, Nakamura M (2018). Establishment of postal audit system in intensity-modulated radiotherapy by radiophotoluminescent glass dosimeters and a radiochromic film. Phys Med.

[13] Murakami N, Okamoto H, Isohashi F (2015). A surveillance study of intensity-modulated radiation therapy for postoperative cervical cancer in Japan. J Radiat Res.

[14] Toita T, Ohno T, Kaneyasu Y (2010). A consensus-based guideline defining the clinical target volume for pelvic lymph nodes in external beam radiotherapy for uterine cervical cancer. Jpn J Clin Oncol.

[15] Murakami N, Norihisa Y, Isohashi F (2016). Proposed definition of the vaginal cuff and paracolpium clinical target volume in postoperative uterine cervical cancer. Pract Radiat Oncol.

[16] Gay HA, Barthold HJ, O'Meara E (2012). Pelvic normal tissue contouring guidelines for radiation therapy: a radiation therapy oncology group consensus panel atlas. Int J Radiat Oncol Biol Phys.

[17] Okamoto H, Murakami N, Carvajal CC (2018). Positional uncertainty of vaginal cuff and feasibility of implementing portable bladder scanner in postoperative cervical cancer patients. Phys Med.

[18] Dice LR (1945). Measures of the amount of ecologic association between species. Ecology.

[19] Huttenlocher DP, Klanderman GA, Rucklidge WJ (1993). Comparing images using the Hausdorff distance. IEEE Trans Pattern Anal Mach Intell.

[20] Fenton PA, Hurkmans C, Gulyban A (2013). Quality assurance of the EORTC 22043-30041 trial in post-operative radiotherapy in prostate cancer: results of the dummy run procedure. Radiother Oncol.

[21] Fairchild A, Langendijk JA, Nuyts S (2014). Quality assurance for the EORTC 22071-26071 study: dummy run prospective analysis. Radiat Oncol.

[22] Schimek-Jasch T, Troost EG, Rücker G (2015). A teaching intervention in a contouring dummy run improved target volume delineation in locally advanced non-small cell lung cancer: reducing the interobserver variability in multicentre clinical studies. Strahlenther Onkol.

[23] Christiaens M, Collette S, Overgaard J (2017). Quality assurance of radiotherapy in the ongoing EORTC 1219-DAHANCA-29 trial for HPV/p16 negative squamous cell carcinoma of the head and neck: results of the benchmark case procedure. Radiother Oncol.

[24] Coskun M, Straube W, Hurkmans CW (2013). Quality assurance of radiotherapy in the ongoing EORTC 22042-26042 trial for atypical and malignant meningioma: results from the dummy runs and prospective individual case reviews. Radiat Oncol.

[25] Abrunhosa-Branquinho AN, Bar-Deroma R, Collette S (2018). Radiotherapy quality assurance for the RTOG 0834/EORTC 26053-22054/NCIC CTG CEC.1/CATNON intergroup trial "concurrent and adjuvant temozolomide chemotherapy in newly diagnosed non-1p/19q deleted anaplastic glioma": individual case review analysis. Radiother Oncol.

[26] Eminowicz G, McCormack M (2015). Variability of clinical target volume delineation for definitive radiotherapy in cervix cancer. Radiother Oncol.

[27] Eminowicz G, Rompokos V, Stacey C (2016). The dosimetric impact of target volume delineation variation for cervical cancer radiotherapy. Radiother Oncol.

[28] Jhingran A, Winter K, Portelance L (2012). A phase II study of intensity modulated radiation therapy to the pelvis for postoperative patients with endometrial carcinoma: radiation therapy oncology group trial 0418. Int J Radiat Oncol Biol Phys.

