# Supplementary material for: Dummy-run for standardizing plan quality of intensity-modulated radiotherapy for postoperative uterine cervical cancer: Japan Clinical Oncology Group study (JCOG1402)
Source: Radiat Oncol. 2019 Jul 29;14:133. doi: 10.1186/s13014-019-1340-y (PMC6664568; doi:10.1186/s13014-019-1340-y)
Supplement: Supplementary file 1 — Figure S1. DVH criteria and the box-plots with a bar of range for 94 treatment plans in two cases: Case 1: (a) and (b), Case 2: (c) and (d). 100% of dose and volume means the prescribed dose of 50.4 Gy and a whole volume of a structure, respectively (DOCX 46 kb) [file 13014_2019_1340_MOESM1_ESM.docx]

**Additional file 1_Figure1**

| (a) | (b) |
| --- | --- |
|  |  |
| (c) | (d) |
|  |  |
| Fig. 1. DVH criteria and the box-plots with a bar of range for 94 treatment plans in two cases: Case 1: (a) and (b), Case 2: (c) and (d). 100% of dose and volume means the prescribed dose of 50.4 Gy and a whole volume of a structure, respectively. | |
