# Supplementary material for: Dummy-run for standardizing plan quality of intensity-modulated radiotherapy for postoperative uterine cervical cancer: Japan Clinical Oncology Group study (JCOG1402)
Source: Radiat Oncol. 2019 Jul 29;14:133. doi: 10.1186/s13014-019-1340-y (PMC6664568; doi:10.1186/s13014-019-1340-y)
Supplement: Supplementary file 2 — Table S2. Assessed categories in the central review. (DOCX 16 kb) [file 13014_2019_1340_MOESM2_ESM.docx]

**Additional file 2_Table1**

Table 1. Assessed categories in the central review

| Structure | Description |
| --- | --- |
| 1. Nodal CTV | - Cranial margin of common iliac lymph node area is aortic bifurcation. - Bone and muscle is excluded from the CTV. - Adipose connective tissue between the lateral surface of the vertebral body and psoas muscle is included in the CTV. - Caudal margin of the external iliac lymph node area is the superior aspect of the femoral head. - Caudal margin of the obturator lymph node area is the superior part of the obturator foramen. - Caudal margin of the presacral lymph node area is the lower level of S2 or cranial section of piriform muscle. |
| 1. Vaginal cuff CTV | - Cranial margin of the CTV vaginal cuff is 1-1.5 cm cranial from the most cranial vaginal marker/gauze. - Caudal margin of the CTV vaginal cuff is 3-4 cm caudal from the most cranial vaginal marker/gauze or at lowest level of obturator foramen, whichever is lower. - Anterior margin of the CTV vaginal cuff is the posterior border of the bladder or retropubic pad of fat. - Lateral margin of the CTV vaginal cuff is the medial edge of the internal obturator, piriformis, coccygeus, iliococcygeus, or puborectalis muscle; the ischiorectal fossa should be excluded from the CTV vaginal cuff. - The posterior margin of the CTV vaginal cuff is the anterior part of the mesorectal fascia or serous surface of anterior wall of the rectum. |
| 1. PTV margin | - Isotropic PTV margin of 0.5 cm for CTV_LN - Anisotropic PTV margin of RL 0.5, SI 1.0, and AP 1.5 cm for CTV_vagina cuff |
| 1. Rectum | - Cranial margin is the lowest part of the sacroiliac joint. - Caudal margin is the superior part of the anal canal. |
| 1. Bowel bag | - Cranial margin is 2 cm cranial from the most superior part of the PTV. - Caudal margin is the lowest part of the bowel. - Bladder should be excluded from the bowel bag. |
| 1. Pelvic bone | - Cranial/caudal margin is 2 cm from the most superior/inferior part of the PTV. - Bone marrow and intervertebral disc are included. - Lumber and sacral vertebrae, iliac bone, ischial bone, and femur are included. |
| 1. Dose   distribution | - A dose level of 95% covers PTV without cold spot, particularly inside the vaginal cuff. - A dose level of 105% should be avoided in the bowel. - The bowel and bladder should be spared at the dose level of 40 Gy. |
| 1. DVH | - Dose and dose-volume criteria for each structure. |
